# Clinical and mechanistic evidence on metabolic effects of oat β-glucan, rice bran, and unripe banana flour: a systematic review with mechanism-informed synthesis

**DOI:** 10.1017/jns.2026.10127

**Published:** 2026-07-22

**Authors:** Alfina Nur Isyrofi, Widya Rahmawati, Xu-Feng Huang, Yuanyi Xie, Warangkana Srichamnong, Dian Handayani

**Affiliations:** 1 Master of Nutrition Science Study Program, Faculty of Health Sciences, Universitas Brawijaya, Indonesia; 2 Nutrition Department, Faculty of Health Sciences, Universitas Brawijaya, Indonesia; 3 University of Wollongong School of Medical Indigenous and Health Sciences, Australia; 4 Institute of Nutrition, Mahidol University Institute of Nutrition, Thailand

**Keywords:** Dietary fibre, Metabolic syndrome, Oat β-glucan, Rice bran, Unripe banana flour

## Abstract

Dietary fibre improves metabolic health, yet comparative evidence across different fibre sources remains unclear. This systematic review evaluates metabolic effects of oat β-glucan, rice bran, *and* unripe banana flour in adults, assessing glycaemic, lipid, and mechanistic effects, synthesising their physiological pathways across distinct fibre types (viscosity-driven, phytochemical-mediated, and resistant starch fermentation mechanisms). Following PRISMA 2020 guidelines, PubMed, Scopus, and Web of Science were searched up to June 2025. A total of 602 records were identified, and after removing 146 duplicates, 456 were screened. Forty-four full texts were assessed, and 15 studies were included. Risk of bias was evaluated using RoB 2.0 and Newcastle–Ottawa. Outcomes included glycaemic markers, insulin response, lipid profile, blood pressure, body composition, and mechanistic biomarkers. Studies (*n* = 15; sample sizes 10–154; durations 3 days–12 weeks) demonstrated heterogeneous dosing and formulations. *Oat β-glucan* consistently improved postprandial glucose, insulin AUC, and LDL-C, with modest effects on waist circumference and blood pressure. *Rice bran improved blood lipids consistently, while glycaemic effects were mixed*. *UBF resistant starch was associated with reduction in fasting glucose, HOMA-IR, and body weight, although findings are based on small, short-duration trials*. However, variability in study design and limited mechanistic assessments restricted cross-fibre synthesis. The available evidence suggests modest improvements in glycaemic and lipid outcomes. Differences in responsiveness likely reflect variations in fibre structure, viscosity, and fermentability, suggesting complementary physiological pathways. Standardised dosing, longer interventions, and mechanistic biomarkers – including SCFA profiles, incretin response, and bile acid signalling – are needed to clarify inter-individual variability and guide fibre-based interventions.

## Graphical abstract

**Figure:**
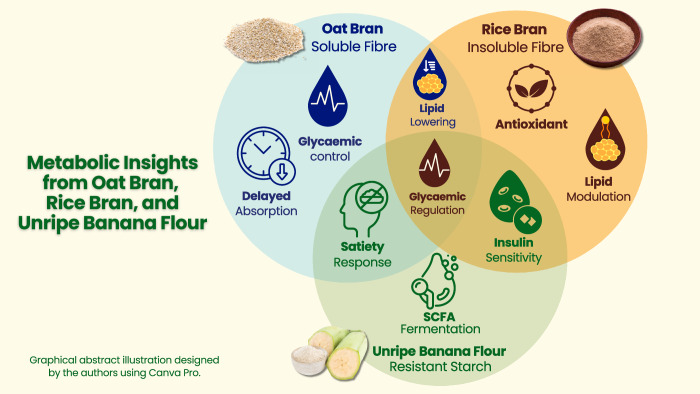

## Introduction

Metabolic syndrome (MetS) represents a major global health challenge characterised by the coexistence of central obesity, dyslipidaemia, elevated blood pressure, and impaired glucose regulation, which collectively heighten the risk of type 2 diabetes mellitus (T2DM) and CVD.^(1,2)^ The prevalence of MetS has risen markedly in both developed and developing nations, driven by dietary westernisation, reduced physical activity, and urbanisation-related lifestyle changes.^(2,3)^ In Southeast Asia, where traditional diets are rapidly being replaced by energy-dense and fibre-poor foods, this upward trend is particularly alarming.^(4)^

Dietary modification remains a cornerstone of non-pharmacological strategies to mitigate the metabolic and inflammatory disturbances associated with MetS. Among nutritional components, dietary fibre has emerged as a pivotal modulator of metabolic health. Soluble and fermentable fibres, such as β-glucan, psyllium, and inulin, have demonstrated the capacity to attenuate postprandial glycaemic responses, reduce total cholesterol (TC) and LDL-C, and improve insulin sensitivity.^(5,6)^ These effects are mediated through mechanisms including delayed gastric emptying, increased satiety, altered bile acid metabolism, and the fermentation of indigestible carbohydrates into SCFA, which exert systemic metabolic benefits and anti-inflammatory actions.^(7)^

The selection of oat bran, rice bran, and unripe banana flour in this systematic review is grounded in their classification as three distinct functional dietary fibre categories with complementary mechanisms relevant to metabolic health. Staple foods commonly consumed in Asian populations such as oats, rice bran, and unripe banana flour, constitute culturally relevant sources of bioactive and fermentable fibres. Oats provide β-glucan, a viscous polysaccharide that can lower serum cholesterol and modulate glycaemic response.^(8)^ Rice bran is rich in arabinoxylans, γ-oryzanol, and other phenolic compounds that improve lipid metabolism and oxidative balance.^(9,10)^ Unripe banana flour, characterised by its high resistant starch content (RS2), influences gut microbial composition and SCFA production, thereby supporting glucose and lipid homeostasis^(11)^ These fibre sources not only differ in their physicochemical properties and fermentability but also in their mechanisms of metabolic action. Taken together, these fibre sources differ fundamentally in their physicochemical properties and biological mechanisms, including viscosity-mediated glucose regulation (β-glucan), phytochemical-driven lipid modulation (rice bran), and microbiota-mediated fermentation pathways (resistant starch). This mechanistic diversity provides a basis for comparative evaluation across fibre types, which remains underexplored in the current literature.

Despite a substantial body of evidence on individual fibre types, most randomised controlled trials (RCTs) and systematic reviews have predominantly focused on single fibres (e.g. β-glucan, rice bran, or resistant starch), often with relatively small sample sizes and short intervention durations, which may limit the generalizability of findings.^(8,12)^ There remains a lack of integrative synthesis that directly compares the distinct physiological mechanisms underlying different fibre types, particularly how differences in viscosity, fermentability, and bioactive composition translate into differential metabolic outcomes. Variability in fibre dosage, processing methods, and food matrix further contributes to heterogeneity in reported clinical effects.^(13)^ For instance, physicochemical properties such as the molecular weight and solubility of β-glucan or the retrogradation characteristics of resistant starch can markedly influence metabolic efficacy.^(14)^ Addressing this gap is critical to advance mechanism-based dietary recommendations rather than fibre-specific conclusions.

Given these research gaps, a comprehensive synthesis of the available human evidence is required to delineate the relative metabolic effects of diverse functional fibres. This systematic review aims to provide a mechanism-informed comparative narrative synthesis of clinical and mechanistic evidence on oat β-glucan, rice bran, and unripe banana flour, with emphasis on how differences in physicochemical properties, viscosity, and fermentation profiles influence metabolic outcomes. The review focuses on glycaemic control, lipid modulation, and blood pressure regulation, while also examining fibre dosage, food matrix interactions, and underlying biological mechanisms relevant to MetS prevention and management. By integrating clinical outcomes with mechanistic pathways, this review seeks to provide a more comprehensive understanding of how distinct fibre types contribute to cardiometabolic health beyond fibre-specific effects.

## Materials and methods

### Study design and reporting guideline

This systematic review was conducted in accordance with the Preferred Reporting Items for Systematic Reviews and Meta-Analyses (PRISMA) 2020 statement.^(15)^ The review protocol was developed a priori to define the study objectives, eligibility criteria, search strategy, and data extraction procedures. The review protocol was submitted to and registered in the PROSPERO international prospective register of systematic reviews (CRD420251061052). The review aimed to synthesise recent human studies on the metabolic effects of three dietary fibre sources – oat β-glucan, rice bran (including defatted rice bran and rice bran oil (RBO)), and unripe (green) banana flour – on glycaemic, lipid, and cardiovascular outcomes.

### Search strategy and information sources

A comprehensive search was undertaken in PubMed (MEDLINE), Scopus, and Web of Science. The search covered records indexed from 1 January 2014 to 30 June 2025. The 10-year inclusion window (2014–2025) was deliberately applied to prioritise studies reflecting recent advances in fibre formulation, food matrix integration, and clinical translation. Earlier trials on β-glucan and resistant starch established foundational physiological effects; however, more recent studies better capture contemporary intervention designs, including optimised molecular characteristics, improved delivery formats (e.g. enriched foods vs isolated supplements), and real-world dietary applications. This restriction was therefore intended to enhance the clinical relevance and comparability of findings across fibre types, rather than to exclude earlier evidence.

Search terms were developed using the PICO framework, combining controlled vocabulary (MeSH) with free-text terms and their synonyms to ensure comprehensive coverage of relevant studies. The representative PubMed search string was:

(‘oat β-glucan’ OR ‘oat bran’ OR ‘rice bran’ OR ‘defatted rice bran’ OR ‘unripe banana’ OR ‘banana flour’ OR ‘resistant starch’ OR ‘soluble fibre’ OR ‘dietary fibre’) AND (‘metabolic syndrome’ OR ‘insulin resistance’ OR ‘dyslipidemia or dyslipidaemia’ OR ‘obesity’ OR ‘hypertension’) AND (‘randomised controlled trial’ OR ‘RCT’)

Equivalent strings, adapted for database syntax, were applied to Scopus and Web of Science based on this primary PubMed formulation.

### Study selection and screening procedure

All records identified were exported to EndNote X9 for duplicate removal. Title and abstract screening were performed independently by two reviewers (Reviewer A and Reviewer B). Full-text articles were retrieved for records judged potentially eligible. Two reviewers independently assessed full texts against prespecified inclusion and exclusion criteria. Discrepancies at any stage were resolved by discussion; a third reviewer adjudicated unresolved conflicts.

A PRISMA flow diagram was constructed to document the number of records identified, screened, excluded (with reasons at full-text stage), and included. Common reasons for exclusion at full text included: study design not eligible (e.g. cross-sectional), fibre source not one of the three target materials, no relevant metabolic outcomes reported, or population outside predefined eligibility.

### Eligibility criteria

**Population.** Population included adults (≥18 years) with MetS or at least one major MetS component (overweight/obesity, impaired fasting glucose, type 2 diabetes, dyslipidaemia, or hypertension). Studies in children, pregnant women, individuals with type 1 diabetes, or specialised clinical populations with unrelated advanced disease were excluded.

**Intervention.** Human consumption of oat β-glucan/oat bran, rice bran, defatted rice bran, or RBO, unripe (green) banana flour (resistant starch type 2), delivered as supplements, fortified foods, or whole-food preparations.

**Comparison.** Placebo, low-fibre/standard diet, or non–fibre-enriched control food.

**Outcomes.** Studies reporting at least one of the following: fasting glucose, postprandial glucose/AUC, HbA1c, insulin, HOMA-IR/QUICKI, TC, LDL-C, HDL-C, TG, blood pressure, body weight, BMI, waist circumference, secondary physiological markers (gut hormones, microbiota, SCFA, inflammation).

**Study design.** RCTs (parallel or crossover; double-blind, single-blind, or open-label) were prioritised. Non-randomised intervention studies were also eligible when random allocation was not feasible but comparative inference remained possible. These studies were synthesised narratively and assessed using the Newcastle–Ottawa Scale (NOS). Effects were interpreted narratively, with RCTs weighted more heavily than non-randomised interventions. Cohort studies, cross-sectional designs, case reports, and reviews were excluded.

Language was restricted to English because of resource limitations for translation. If critical non-English trials were identified by title/abstract, authors were contacted to request English abstracts or data; none were included in this review.

### Data extraction

Data extraction was undertaken independently by two reviewers using a standardised form. Extracted items included:

- author, year, country, and study design;
- sample size, participant characteristics (age, sex, baseline BMI, health status);
- intervention details (fibre source, dose, molecular characteristics where reported, formulation and food matrix, frequency, duration);
- comparator;
- primary and secondary outcomes with units and time points;
- statistical analyses, effect estimates, CI and *p*-values;
- adverse events, tolerability and attrition rates.

All data extraction was performed independently by two reviewers, with discrepancies resolved by discussion or consultation with a third reviewer when necessary. When required, authors were contacted to obtain missing data or clarify methods. Continuous outcomes presented as medians were converted to means where justified using established methods.^(16)^ Where change-from-baseline standard deviations were not reported, these were imputed following Cochrane Handbook guidance using available variance data or correlation coefficients.

Extracted data were organised into Table 1 (Primary study characteristics), Tables 4–7 (Fibre-level comparative synthesis and outcome details).

**Table 1.** Summary of included studies by fibre type, design, population, intervention dose and duration, outcomes, and reported side effects Table 1 long description.

Author (Year)	Sample (*n*; population)	Intervention (dose) vs Control	Duration	Key outcomes (Δ / % / *p*-value)
**Oat β-glucan**				
Steinert et al. 2016^(18)^	*n* = 10; healthy/overweight	Oat β-glucan (0.9–5.3 g) vs control	Acute	Glucose AUC: −4.35% per g (*p* < 0.05); peak glucose: −6.57% per g (*p* < 0.05)
Hjorth et al. 2025^(19)^	*n* = 194; adults at T2DM risk	β-glucan bread vs control bread	16 weeks	Fasting glucose: ↓; HbA1c: ↓ (trend)
AlFaris and Ba-Jaber 2020^(20)^	*n* = 78; obese T2DM women	Oat bran (10 g/day) + diet vs diet	12 weeks	LDL-C: −20% (*p* < 0.05); TC: −8.3% (*p* < 0.05); BMI: −3–4%
García-Cordero et al. 2023^(21)^	*n* = 29; overweight/obese	β-glucan (2.5 g/day) vs control	8 weeks	VLDL-C: ↓ (*p* < 0.05); DBP: ↓ (*p* < 0.05)
Leão et al. 2019^(22)^	*n* = 154; MetS	Oat bran (40 g/day) + low-calorie diet vs diet alone	6 weeks	BMI: ↓ (*p* < 0.05); WC: ↓ (*p* < 0.05); BP: ↓ (*p* < 0.05); TG: ↓ (*p* < 0.05); glucose: ↓ (*p* < 0.05); HDL-C: − (*p* = 0.025); between-group difference: not significant
Xue et al. 2021^(52)^	*n* = 50; hypertension	Oat bran (30 g/day) vs control diet	12 weeks	SBP: ↓ (*p* < 0.05); DBP: ↓ (*p* < 0.05); microbiota diversity: ↑
**Rice bran**				
Mahdavi-Roshan et al. 2024 ^(10)^	*n* = 50; MetS	Rice bran oil (30 g/day) vs control	8 weeks	LDL-C: ↓ (*p* < 0.001); HDL-C: ↑ (*p* < 0.05); MDA: ↓
Lin et al. 2020^(23)^	*n* = 45; mixed population	Rice bran (40 g/day) vs control	8 weeks	WC: ↓ (*p* < 0.05); BP: ↓ (*p* < 0.05); glucose: ↓ (*p* < 0.05); HbA1c: ↓ (*p* < 0.05); TG: ↓ (*p* < 0.05)
Saphyakhajorn et al. 2022^(24)^	*n* = 61; overweight	Defatted rice bran (30 g/day) vs placebo	12 weeks	HbA1c: −3.6% (*p* < 0.05); BP: −4–5% (*p* < 0.05)
Prasertsri et al. 2022^(25)^	*n* = 35; aged 60 to 76 years with prehypertension	Rice bran oil capsules vs control	8 weeks	Oxidative stress markers: ↓; CVD markers: ↓
Ng et al. 2025^(53)^	*n* = 56; healthy adults	Rice bran bread vs control	4 weeks per phase (2-week washout)	Microbiota diversity: ↑
**Unripe banana flour (RS)**				
Sardá et al. 2016^(26)^	*n* = 22; healthy males	UBF (8 g, 3×/week) vs placebo	6 weeks	Hunger: ↓; PYY: ↑; insulin: ↓; insulin sensitivity: ↑
Silva et al. 2014^(27)^	*n* = 25; overweight women	GBF (20 g/day) vs control	45 days	BP: ↓; glucose: ↓ (*p* = 0.032)
Costa et al. 2019^(28)^	*n* = 113; prediabetes/T2DM	Banana biomass vs control	24 weeks	Glucose: ↓; body weight: ↓; lipid profile: improved
de Oliveira Lomeu et al. 2021 ^(29)^	*n* = 60; overweight women	UBF beverage vs control	8 weeks	Body weight: ↓; WC: ↓; fasting glucose: ↓

**Table 2.** Risk of bias assessment of included studies: randomised controlled trials (RoB 2.0 assessment) Table 2 long description.

Study (Author, Year)	Fibre type	Study design	Randomisation process	Deviations from intended interventions	Missing outcome data	Measurement of the outcome	Selection of the reported result	Overall risk of bias
Steinert et al. 2016 ^(18)^	Oat β-glucan	RCT	Low risk	Some concerns (open-label)	Low risk	Low risk	Some concerns	Some concerns
Hjorth et al. 2025 ^(19)^	Oat β-glucan	RCT	Low risk	Low risk	Low risk	Low risk	Low risk	Low
AlFaris and Ba-Jaber, 2020^(20)^	Oat β-glucan	RCT	Low risk	Low risk	Low risk	Low risk	Low risk	Low
García-Cordero et al. 2023^(21)^	Oat β-glucan	RCT	Low risk	Low risk	Low risk	Low risk	Low risk	Low
Leão et al. 2019^(22)^	Oat β-glucan	RCT	Low risk	High risk (unprespecified HDL-C endpoint)	Low risk	Low risk	High risk	High
Xue et al. 2021^(52)^	Oat β-glucan	RCT	Low risk	Some concerns (unblinded)	Low risk	Low risk	Some concerns	Some concerns
Mahdavi-Roshan et al. 2024^(10)^	Rice bran	RCT	Low risk	Low risk	Low risk	Low risk	Low risk	Low
Lin et al. 2020^(23)^	Rice bran	RCT	Low risk	Low risk	Low risk	Low risk	Low risk	Low
Saphyakhajorn et al. 2022^(24)^	Rice bran	RCT	Low risk	Low risk	Low risk	Low risk	Low risk	Low
Prasertsri et al. 2022^(25)^	Rice bran	RCT	Low risk	Low risk	Low risk	Low risk	Low risk	Low
Ng et al. 2025^(53)^	Rice bran	RCT	Low risk	Some concerns	Low risk	Low risk	Some concerns	Some concerns
Hoffmann Sardá et al. 2016^(26)^	Unripe banana flour	RCT	Low risk	Some concerns (small sample, unblinded)	Low risk	Low risk	Some concerns	Some concerns
Costa et al. 2019 ^(28)^	Unripe banana flour	RCT	Low risk	Some concerns (open-label)	Low risk	Low risk	Some concerns	Some concerns
de Oliveira Lomeu et al. 2021^(29)^	Unripe banana flour	RCT	Low risk	Some concerns (blinding unclear)	Low risk	Low risk	Some concerns	Some concerns

*Notes*: RoB 2.0 domains include: randomisation process; deviations from intended interventions; missing outcome data; measurement of the outcome; and selection of the reported result.

Judgements are categorised as low risk, some concerns, or high risk according to Cochrane guidelines.

The Newcastle–Ottawa Scale (NOS) evaluates non-randomised studies across three domains: selection, comparability, and outcome (maximum score: 9 stars).

Studies scoring ≥7 stars were considered moderate-to-high quality (low risk of bias), while scores <7 indicate higher risk.

**Table 3.** Risk of bias assessment of included studies: non-randomised study (Newcastle–Ottawa scale assessment) Table 3 long description.

Study (Author, Year)	Fibre type	Study design	Selection (max 4)	Comparability (max 2)	Outcome (max 3)	NOS score (0–9)	Overall risk of bias
Silva et al. 2014^(27)^	Unripe banana flour	Non-randomised intervention	★★★	★★	★★	7/9	Moderate

*Notes*: RoB 2.0 domains include: randomisation process; deviations from intended interventions; missing outcome data; measurement of the outcome; and selection of the reported result.

Judgements are categorised as low risk, some concerns, or high risk according to Cochrane guidelines.

The Newcastle–Ottawa Scale (NOS) evaluates non-randomised studies across three domains: selection, comparability, and outcome (maximum score: 9 stars).

Studies scoring ≥7 stars were considered moderate-to-high quality (low risk of bias), while scores <7 indicate higher risk.

**Table 4.** Randomised controlled trials on oat β-glucan and metabolic outcomes (included studies published between 2014–2025) Table 4 long description.

Author (Year)	Country	Population	Study design	Sample size	Duration	dose	Fibre source	Primary outcomes	Risk of bias
Steinert et al. 2016 ^(18)^	Canada	Healthy adults	Randomised crossover (acute)	10	Single meal	0–5.3 g	Oat bran (OatWell®)	Postprandial glycaemic response (AUC), peak glucose	Some concerns
Hjorth et al. 2025 ^(19)^	Sweden/Germany/Norway	Adults at risk of T2DM	Parallel RCT	194	16 weeks	≥3 slices/day (6 g β-glucan day), 6 days/week	β-glucan-enriched bread	Fasting glucose, HbA1c	Low
AlFaris & Ba-Jaber 2020^(20)^	Saudi Arabia	Women with T2DM	Parallel RCT	78	12 weeks	10 g/day	Oat bran ± olive oil	LDL-C, TC, BMI	Low
García-Cordero et al. 2023^(21)^	Spain	Overweight/obese adults	Randomised crossover, blinded	29	8 weeks	2.5 g/day	β-glucan capsule	VLDL-C, DBP	Low
Leão et al. 2019^(22)^	Brazil	Adults with MetS	Pragmatic randomised controlled trial (open-label, parallel)	154	6 weeks	40 g/day	Oat bran (≈3 g β-glucan)	No additional effect on MetS remission vs control; ↓ BMI, WC, BP, TG, glucose in both groups; ↓ HDL-C in intervention group	High
Xue et al. 2021^(52)^	China	Adults with MetS	Open-label RCT	50	12 weeks	30 g/day	Oat bran	BMI, BP, TG, fasting glucose	Some concerns

*Note:* Only studies published between 2014 and 2025 were included, in accordance with the predefined search strategy. Earlier studies were excluded by design to ensure relevance to recent advances in functional fibre interventions.

**Table 5.** Randomised controlled trials on rice bran and metabolic outcomes (included studies published between 2014–2025) Table 5 long description.

Author (Year)	Country	Population	Study design	Sample size	Duration	Dose	Fibre source	Primary outcomes	Risk of bias
Mahdavi-Roshan et al. 2024^(10)^	Iran	Adults with MetS	Open-label RCT	50	8 weeks	30 g/day	Rice bran oil	Lipids, HOMA-IR, oxidative stress	Moderate
Lin et al. 2020 ^(23)^	China/Taiwan	Adults (mixed metabolic status)	Randomised trial	45	8 weeks	40 g/day	Refined rice bran	WC, BP, glucose, HbA1c, TG	Not clearly reported
Saphyakhajorn et al. 2022^(24)^	Thailand	Overweight/obese adults	Double-blind RCT	61	12 weeks	30 g/day	Defatted rice bran	HbA1c, lipids, BP	Low
Prasertsri et al. 2022^(25)^	Thailand	Older adults	RCT	35	Several weeks	1–2 g/day	Rice bran oil	Oxidative stress, CVD markers	Not clearly reported
Ng et al. 2025^(53)^	New Zealand	Healthy adults	Crossover RCT	56	4 weeks per phase (2-week washout)	3 slices/day (females) or 4 slices/day (males)	Rice bran bread	Gut microbiota composition, transit time, cardiovascular risk, gut discomfort	Some concerns

*Note:* Only studies published between 2014 and 2025 were included, in accordance with the predefined search strategy. Earlier studies were excluded by design to ensure relevance to recent advances in functional fibre interventions.

**Table 6.** Studies on unripe banana flour and metabolic outcomes (included studies published between 2014–2025. RCTs and non-randomised studies) Table 6 long description.

Author (Year)	Country	Population	Study design	Sample Size	Duration	Dose	Fibre Source	Primary Outcomes	Risk of Bias
Sardá et al. 2016 ^(26)^	Brazil	Healthy adults	Double-blind RCT	22	6 weeks	∼8 g/dose (3×/week)	Unripe banana flour (RS2)	Satiety, insulin sensitivity	Low
Silva et al. 2014^(27)^	Brazil	Women with MetS	Intervention trial	25	45 days	20 g/day	Green banana flour	BP, fasting glucose	Some concerns
Costa et al. 2019 ^(28)^	Brazil	Pre-diabetes/T2DM	RCT	113	24 weeks	40 g/day green banana biomass (approx. 4.5 g RS)	Banana biomass	Glucose, weight, lipids	Some concerns
de Oliveira Lomeu et al. 2021^(29)^	Brazil	Overweight women	Double-blind RCT	60	8 weeks	Not reported	UBF + cocoa	Anthropometric, biochemical markers	Moderate

*Note*: Only studies published between 2014 and 2025 were included, in accordance with the predefined search strategy. Earlier studies were excluded by design to ensure relevance to recent advances in functional fibre interventions.

Non-randomised studies are included for completeness and interpreted with caution.

**Table 7.** Comparative summary of metabolic effects across dietary fibre types (oat β-glucan, rice bran, and unripe banana flour) based on included randomised controlled trials Table 7 long description.

Fibre type	Glycaemic outcomes	Anthropometric outcomes	Lipid outcomes	Blood pressure	Oxidative stress / inflammation	Mechanistic interpretation	Strength of evidence
**Oat β-glucan** (oat bran / enriched foods)	Reductions in postprandial glucose responses are consistently reported, with smaller and less consistent effects on fasting glucose and HbA1c across longer interventions	Modest reductions in body weight and waist circumference, primarily in studies combined with dietary modification	Reductions in total cholesterol and LDL-C are consistently observed; effects on HDL-C and TGs are variable	Small reductions in SBP/DBP reported in several trials	Limited and inconsistent effects on inflammatory markers; some studies report microbiota modulation	Increased intestinal viscosity delays gastric emptying and glucose absorption; bile acid binding contributes to cholesterol lowering; partial fermentation may contribute to SCFA production	*n* ≈ 6 RCTs; consistency: high for postprandial glycaemia and LDL-C; moderate for other outcomes
**Rice bran** (defatted, refined, and oil forms)	Modest reductions in fasting glucose and HbA1c reported in some trials; findings are variable across populations	Minimal or no consistent effects on body weight; small reductions in waist circumference reported in some studies	Improvements in total cholesterol and LDL-C, with some increases in HDL-C, particularly in rice bran oil interventions	Small but significant reductions in blood pressure reported in several studies	More consistent improvements in oxidative stress markers (e.g. MDA, antioxidant capacity); limited effects on CRP	Combined effects of dietary fibre and bioactive compounds (γ-oryzanol, tocotrienols, phytosterols) influencing lipid metabolism, oxidative stress, and inflammation; limited contribution of fermentation relative to other fibres	*n* ≈ 5 RCTs; consistency: moderate for lipid and BP outcomes; mixed for glycaemia
**Unripe banana flour (resistant starch, RS2)**	Improvements in fasting glucose and insulin sensitivity indices (e.g. HOMA-IR) are reported; postprandial responses show modest attenuation	Modest reductions in appetite and, in some studies, body weight or waist circumference	Effects on lipid profile are generally small and inconsistent	Limited and population-specific effects on blood pressure	Evidence suggests modulation of satiety hormones (e.g. PYY, GLP-1); limited data on inflammatory markers	Acute glycaemic effects are primarily related to reduced digestibility and delayed carbohydrate absorption, while longer-term improvements in insulin sensitivity are mediated by colonic fermentation and SCFA production	*n* = 3 RCTs (+1 non-randomised study); consistency: moderate for insulin sensitivity and satiety; limited for other outcomes

*Notes:* Outcomes represent synthesised findings across included randomised controlled trials and should not be interpreted as pooled effect sizes.

FPG = fasting plasma glucose; PPG = postprandial glucose; HbA1c = glycated haemoglobin; SBP/DB*P* = systolic/diastolic blood pressure.

Strength of evidence reflects number of RCTs, consistency of findings, and study design characteristics.

Mechanistic interpretations are based on convergent evidence from included trials and supporting literature.

### Risk of bias and certainty of evidence

Randomised trials were assessed using the Cochrane Risk of Bias 2.0 (RoB 2) tool,^(17)^ covering five domains: randomisation, deviations from intended interventions, missing outcome data, outcome measurement, and selective reporting. Each domain was rated as low risk, some concerns, or high risk, with an overall trial-level judgment assigned.

Non-randomised studies were evaluated using the NOS, assessing selection, comparability, and outcome ascertainment. Studies scoring ≥7 stars were considered high quality.

Two reviewers independently conducted RoB 2 and NOS assessments, resolving discrepancies through discussion or consultation with a third reviewer. Summary judgments for the risk of bias of the included randomised trials and non-randomised studies are presented in and Table 3, respectively. These quality assessments informed our sensitivity analyses; wherein high-risk studies were evaluated to examine the overall robustness of the synthesised findings.

### Data synthesis and statistical analysis

A narrative synthesis was performed for all included studies and structured by fibre type and outcome domain. Meta-analysis was planned when at least three clinically and methodologically homogeneous trials addressed the same outcome for a given fibre type. A meta-analysis was not feasible due to incompatible outcome measures, non-standardised dosing, and insufficiently reported variance data in several trials. Random-effects meta-analysis (DerSimonian-Laird) was specified a priori to account for between-study heterogeneity. Effect estimates for continuous outcomes were pooled as mean differences (MD) or standardised mean differences (SMD) with 95% CI. Heterogeneity was quantified by the *I* ^2^ statistic and Cochran’s *Q* test. Subgroup analyses were prespecified for dose categories, intervention duration (<8 vs ≥8 weeks), baseline risk status (healthy vs MetS/diabetic), and food matrix (isolated supplement vs whole food). Sensitivity analyses were planned by excluding studies at high risk of bias.

Publication bias was investigated using funnel plots and Egger’s regression test when ≥10 studies were available for a particular meta-analysis. Statistical analyses were conducted using Review Manager (RevMan 5.4) and Stata 17 (StataCorp).

When meta-analysis was not possible due to clinical heterogeneity or insufficient data, results were synthesised narratively with effect direction and magnitude described and tabulated.

### Terminology, consistency and reporting decisions

To address reviewer concerns about inconsistent terminology, the term *fibre* (British English) is used throughout the manuscript. Synonyms encountered in primary studies – fibre, green banana, unripe banana – are reported in data extraction but outcomes and narrative synthesis use standardised terminology: unripe (green) banana flour *for banana-derived resistant starch and* defatted rice bran *or* RBO as appropriate. This approach ensured consistent terminology across the manuscript, addressing potential ambiguities highlighted during peer review.

### Ethical considerations and transparency

**Figure 1.**
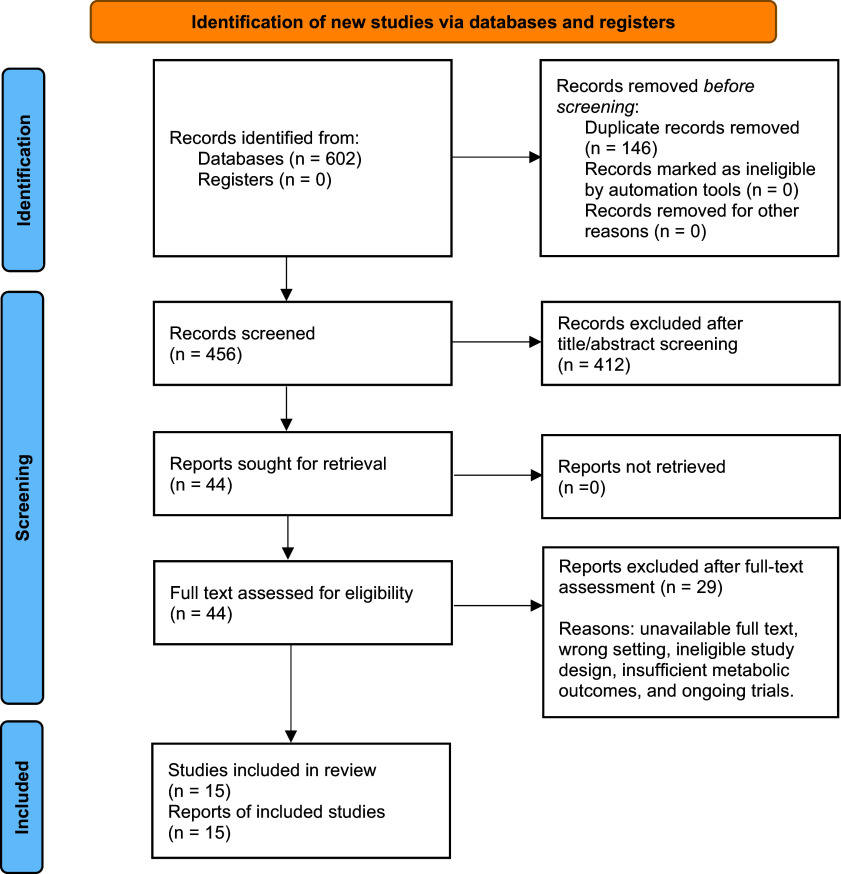
Figure 1 long description. PRISMA flow diagram.

Ethical approval was not required because the study synthesised previously published, anonymised data. The review adhered to transparency principles; the search strategy, data extraction templates, and risk-of-bias judgements fully presented within the main text and tables. Study selection and reasons for exclusion at full-text screening are detailed in the PRISMA flow diagram (Figure 1).

## Results

### Study selection and characteristics

The systematic search identified 602 records from electronic databases (PubMed, Scopus, and Web of Science). After removal of 146 duplicates, 456 records were screened at the title and abstract level, of which 412 were excluded for not meeting the predefined eligibility criteria. A total of 44 full-text articles were retrieved and assessed for eligibility. Twenty-nine full-text reports were excluded for the following reasons: unavailable full text (*n* = 6), wrong setting or unrelated intervention context (*n* = 8), ineligible study design (*n* = 6), insufficient metabolic outcomes (*n* = 6), and ongoing trial publications (*n* = 3). Ultimately, 15 studies met all inclusion criteria and were included in the final systematic review.

A total of 15 studies meeting all eligibility criteria were included in this systematic review (Figure 1). These studies summarised in Table 1, including fibre source, design, population, intervention dose and duration, metabolic outcomes, and side effects. The included studies encompassed diverse populations, including healthy adults, overweight or obese individuals, and participants with MetS, hypertension, or type 2 diabetes. Interventions varied widely in fibre source, dose, and duration, ranging from 3 days to 12 weeks with sample sizes from 10 to 154 participants. Of the 15 included studies, 14 were RCT and 1 was a non-randomised intervention. The studies examined three major fibre types: oat β-glucan (*n* = 6), rice bran (*n* = 5), and unripe banana flour (*n* = 4; including 3 randomised and 1 non-randomised study). Effects from RCTs were given more weight in the narrative synthesis, while the non-randomised study was interpreted cautiously.

Risk of bias assessment using RoB 2.0 and the Newcastle–Ottawa Scale (NOS) is presented in Table 2. Most studies showed low or moderate risk of bias, with some concerns related to lack of blinding and incomplete outcome reporting.

### Outcomes by fibre type

#### Oat β-glucan

Six studies investigated oat β-glucan interventions, including enriched breads, oat bran supplements, and high–molecular weight oat β-glucan preparations. Glycaemic outcomes were generally improved across studies. Steinert et al. (2016) demonstrated a clear dose-dependent reduction in postprandial glucose AUC following consumption of 0.9–5.3 g of β-glucan, with each gram reducing glucose AUC by 4.35% and peak glucose by 6.57%.^(18)^ Long-term consumption of β-glucan-enriched bread was associated with improved fasting glycaemia and HbA1c trends in adults at elevated risk for type 2 diabetes.^(19)^

Lipid outcomes also showed generally positive trends. Significant reductions in LDL-C and TC were reported among participants following a low energy diet with oat bran supplementation.^(20)^ Reductions in VLDL-C and diastolic blood pressure were also observed after eight weeks of supplementation.^(21)^

Microbiota modulation was documented in multiple studies, particularly increased *Bifidobacterium* and *Spirillum* species in participants consuming oat bran. Taken together, findings across these relatively small and heterogeneous trials suggest multi-pathway metabolic activity involving glycaemic regulation, lipid metabolism, and gut microbiota. In contrast, Leão et al. (2019)^(22)^ reported that the addition of 40 g/day oat bran to a low-calorie diet did not confer additional benefits compared with diet alone, although both groups showed significant improvements in metabolic parameters. Notably, a reduction in HDL-C was observed in the intervention group, indicating a potentially unfavourable lipid response.

A detailed overview of individual RCTs assessing oat β-glucan is presented in Table 4.

#### Rice bran

Five studies investigated rice bran in various forms, including refined rice bran, defatted rice bran, and RBO. Lin et al. (2020)^(23)^ documented improvements in waist circumference, blood pressure, fasting glucose, HbA1c, and TGs after eight weeks of refined rice bran consumption. Similarly, Saphyakhajorn et al. (2022)^(24)^ demonstrated significant reductions in HbA1c and blood pressure after 12 weeks of defatted rice bran supplementation.

RBO showed marked lipid improvements, with Mahdavi-Roshan et al. (2024)^(10)^ reporting significant reductions in TCl and LDL-C and increases in HDL-C. Mechanistic findings suggest the involvement of γ-oryzanol, tocotrienols, and phytosterols in enhancing bile acid excretion and antioxidant capacity.

Oxidative stress improvements were reported in older adults receiving Riceberry RBO,^(25)^ while other studies documented favourable shifts in gut microbiota.

A structured summary of rice bran RCTs is provided in Table 5.

#### Unripe banana flour

Three RCT assessed unripe banana flour (UBF/GBF). One additional non-randomised study was identified and is considered in the narrative interpretation only. Sardá et al. (2016)^(26)^ reported improvements in satiety, reduced hunger, decreased ghrelin AUC, increased PYY, and improved insulin sensitivity. Silva et al. (2014)^(27)^ found improvements in fasting glucose and blood pressure among women with MetS. Costa et al. (2019) and de Oliveira Lomeu et al. (2021)^(28,29)^ documented reductions in body weight, waist circumference, and fasting glucose. The limited number of randomised trials and small sample sizes warrant cautious interpretation of these findings.

These metabolic benefits are likely mediated by the high resistant-starch content of UBF, which modulates SCFA production and incretin hormone release.

A complete summary of banana flour interventions is provided in Table 6.

#### Dose–response and mechanistic insights

Clear dose–response effects were evident for oat β-glucan, particularly for postprandial glycaemic responses.^(18)^ Proposed mechanisms include delayed gastric emptying, increased chyme viscosity, glucose absorption modulation, and microbiota-mediated SCFA production.

Rice bran and RBO effects appear linked to bile acid excretion, antioxidant phytochemicals, and ACE-inhibitory peptides. UBF effects on satiety and insulin sensitivity reflect resistant starch fermentation and hormonal regulation.

Mechanistic interpretations across all fibre types are synthesised in Table 7.

#### Safety and adverse events

Across all included studies, dietary fibre interventions were well tolerated. Minor gastrointestinal discomfort was reported sporadically, mainly with oat β-glucan or banana flour consumption. Only one withdrawal occurred due to gastrointestinal symptoms in an oat bran intervention.^(20)^ No serious adverse events were reported.

## Discussion

This systematic review synthesises evidence from 15 studies, including 14 RCT and 1 non-randomised intervention study, evaluating the metabolic effects of three functionally distinct dietary fibres – oat β-glucan, rice bran, and unripe banana flour – across diverse adult populations. The integrated evaluation highlights complementary physiological mechanisms and provides a mechanism-informed synthesis of fibre-specific metabolic pathways. Unlike prior systematic reviews that primarily focus on single fibre types (e.g. β-glucan, rice bran, or resistant starch), this review integrates clinical outcomes with mechanistic pathways across fibre classes, enabling a more comprehensive understanding of how differences in viscosity, fermentability, and bioactive composition may contribute to metabolic responses.

### Glycaemic control

All three fibre types were associated with improvements in glycaemic outcomes, albeit through distinct mechanisms. Oat β-glucan was consistently associated with reductions in postprandial glycaemic and insulin responses in a dose-dependent manner, mediated by gel formation, delayed gastric emptying, and modulation of intestinal glucose transporters.^(18,19,30)^ Resistant starch from unripe banana flour was associated with improvements in fasting glucose, HOMA-IR, and, to a lesser extent, postprandial glycaemic responses. These effects appear to arise from complementary mechanisms: acute reductions in postprandial glycaemia are more likely driven by the physical presence of resistant starch within the food matrix, which slows carbohydrate digestion and attenuates glucose absorption, whereas longer-term improvements in fasting glucose and insulin sensitivity are primarily mediated by colonic fermentation and subsequent production of SCFA that modulate insulin signalling and glucose metabolism.^(27,31–33)^ Rice bran was associated with modest and relatively consistent reductions in fasting glucose and HbA1c, potentially through antioxidant and anti-inflammatory phytochemicals such as γ-oryzanol and ferulic acid. ^(23,24,34)^ Overall, the evidence indicates a layered effect where β-glucan provides predictable postprandial control, resistant starch contributes to both fasting and postprandial glycaemic regulation through distinct but complementary mechanisms, and rice bran provides additional metabolic support via bioactive compounds.

### Lipid metabolism

Oat β-glucan and rice bran, particularly RBO, were associated with more pronounced lipid-related improvements. β-glucan was consistently associated with reductions in LDL-C, TC, and VLDL-C through increased bile acid excretion and modulation of hepatic cholesterol metabolism, with efficacy influenced by dose and delivery matrix.^(20,21,35–37)^ RBO improved TC, LDL-C, and TGs, while increasing HDL-C, mediated by γ-oryzanol, tocotrienols, and phytosterols, which modulate cholesterol absorption, hepatic synthesis, and inflammation.^(10,25,38–40)^ Unripe banana flour exhibited modest lipid benefits, enhancing HDL-C and oxidative status, likely via increased fat oxidation and SCFA-mediated pathways.^(28,41,42)^ These mechanistic distinctions support tailored fibre interventions to target specific lipid abnormalities.

### Body weight, satiety, and anthropometrics

All three fibres were associated with favourable effects on body weight, waist circumference, and appetite regulation through complementary mechanisms. Resistant starch from unripe banana flour enhanced satiety hormones (PYY, GLP-1) and modulated energy balance through SCFA-mediated signalling.^(27,29,43,44)^ Oat β-glucan slowed gastric emptying and promoted gut hormone secretion, contributing to reduced energy intake and modest weight loss.^(45)^ Rice bran exerted supplementary benefits on anthropometrics via fibre content and bioactive compounds influencing energy metabolism.^(24)^ Taken together, these findings – derived from a limited and heterogeneous evidence base – suggest a multi-mechanistic model in which fibre type, composition, and food matrix influence satiety and anthropometric outcomes.

### Blood pressure and oxidative stress

Modest reductions in blood pressure were observed with oat β-glucan and rice bran, with mechanisms including improved endothelial function, bile acid-mediated pathways, and antioxidant phytochemicals.^(25,46,47)^ Resistant starch may additionally lower BP through gut–vascular interactions, SCFA production, and modulation of sympathetic tone.^(48)^ These complementary pathways suggest potential of combined fibre interventions to improve vascular health.

### Mechanistic integration

Oat β-glucan exerts effects through gel-mediated modulation of nutrient absorption and suppression of intestinal glucose transporters.^(30)^ Rice bran acts via phytochemical-mediated antioxidant and anti-inflammatory pathways.^(49,50)^ Resistant starch from unripe banana flour operates through microbiota-driven fermentation, SCFA production, and modulation of incretin hormones.^(51)^ This comparative mechanistic framework facilitates selection of fibre interventions tailored to individual metabolic phenotypes.

### Clinical and public health implications

The available evidence suggests that incorporating multiple fibre sources into culturally relevant dietary patterns may help target diverse cardiometabolic pathways. β-glucan is optimal for postprandial glycaemic control, rice bran for lipid and antioxidant modulation, and unripe banana flour for satiety and insulin sensitivity. In Southeast Asia, integrating these fibres into staple foods or functional ingredients offers an accessible, scalable, and evidence-based approach for metabolic risk reduction.

### Strengths and limitations

This review is strengthened by rigorous PRISMA methodology, structured risk-of-bias assessment, and fibre-specific narrative synthesis. However, heterogenity in fibre dose, food matrix, intervention duration, and sample sizes precluded meta-analytic pooling. Mechanistic endpoints were variably reported, limiting synthesis of microbiota, SCFA, and bile acid data. Future studies should standardise dosing, intervention duration, and outcome reporting while integrating mechanistic biomarkers to better understand inter-individual variability.

### Conclusion

This review suggests that oat β-glucan, rice bran, and unripe banana flour may be associated with beneficial effects on cardiometabolic health, highlighting distinct and complementary physiological mechanisms that could inform mechanism-based dietary strategies. Oat β-glucan tended to be associated with improvements in glycaemic and lipid profiles, while rice bran may contribute to antioxidant and lipid-regulating effects, and unripe banana flour may support insulin sensitivity and satiety through resistant starch–related mechanisms. Across the included studies, the available evidence suggests that the inclusion of multiple fibre types – not limited to β-glucan – may be considered a complementary dietary approach for improving metabolic outcomes. Despite heterogeneity and methodological limitations in the available studies, these findings suggest that fibre-based interventions may be beneficial for population at risk of MetS.

## Table 1. Long description

The table compares studies on different fiber types, including oat beta-glucan, rice bran, and unripe banana flour. It has 15 rows and 5 columns. The columns are labeled Author (Year), Sample (n; population), Intervention (dose) vs Control, Duration, and Key outcomes (Δ / % / p-value). Each row provides details of a specific study, including the author and year, sample size and population, the intervention and control conditions, the duration of the study, and the key outcomes measured. The table includes studies on various populations such as healthy adults, overweight individuals, and those with specific health conditions like type 2 diabetes mellitus (T2DM) and metabolic syndrome (MetS). The interventions vary in dose and type, and the outcomes include measurements like glucose levels, cholesterol levels, body mass index (BMI), and blood pressure (BP).

Navigate back to Table 1..

## Table 2. Long description

A table assessing the risk of bias in randomized controlled trials of different fiber types. The table has 13 rows and 7 columns. The columns are labeled Study (Author, Year), Fibre type, Study design, Randomisation process, Deviations from intended interventions, Missing outcome data, Measurement of the outcome, Selection of the reported result, and Overall risk of bias. Each row lists a study with its corresponding fiber type, study design, and various risk assessments. Notable trends include several studies with some concerns in the overall risk of bias, particularly those involving oat beta-glucan and unripe banana flour.

Navigate back to Table 2..

## Table 3. Long description

A table assessing the risk of bias in a non-randomised study using the Newcastle-Ottawa scale. The table has six columns: Study (Author, Year), Fibre type, Study design, Selection (max 4), Comparability (max 2), Outcome (max 3), NOS score (0-9), and Overall risk of bias. It contains one row of data. Row 1: Silva et al. 2014, Unripe banana flour, Non-randomised intervention, 3 stars, 1 star, 3 stars, 7/9, Moderate.

Navigate back to Table 3..

## Table 4. Long description

A table comparing characteristics of randomized controlled trials on oat beta-glucan and metabolic outcomes. The table has 7 rows and 9 columns. Column headers are Author (Year), Country, Population, Study design, Sample size, Duration, Fibre dose, Fibre source, Primary outcomes, Risk of bias. Row 1: Author (Year), Steinert et al. 2016; Country, Canada; Population, Healthy adults; Study design, Randomised crossover (acute); Sample size, 10; Duration, Single meal; Fibre dose, 0-5.3 g; Fibre source, Oat bran (OatWell®); Primary outcomes, Postprandial glycaemic response (AUC), peak glucose; Risk of bias, Some concerns. Row 2: Author (Year), Hjorth et al. 2025; Country, Sweden/Germany/Norway; Population, Adults at risk of T2DM; Study design, Parallel RCT; Sample size, 194; Duration, 16 weeks; Fibre dose, ≥3 slices/day (6 g β-glucan day, 6 days/week); Fibre source, β-glucan-enriched bread; Primary outcomes, Fasting glucose, HbA1c; Risk of bias, Low. Row 3: Author (Year), AlFaris & Ba-Jaber 2020; Country, Saudi Arabia; Population, Women with T2DM; Study design, Parallel RCT; Sample size, 78; Duration, 12 weeks; Fibre dose, 10 g/day; Fibre source, Oat bran ± olive oil; Primary outcomes, LDL-C, TC, BMI; Risk of bias, Low. Row 4: Author (Year), García-Cordero et al. 2023; Country, Spain; Population, Overweight/obese adults; Study design, Randomised crossover, blinded; Sample size, 29; Duration, 8 weeks; Fibre dose, 2.5 g/day; Fibre source, β-glucan capsule; Primary outcomes, VLDL-C, DBP; Risk of bias, Low. Row 5: Author (Year), Leão et al. 2019; Country, Brazil; Population, Adults with MetS; Study design, Pragmatic randomised controlled trial (open-label, parallel); Sample size, 154; Duration, 6 weeks; Fibre dose, 40 g/day; Fibre source, Oat bran (≈3 g β-glucan); Primary outcomes, No additional effect on MetS remission vs control; ↓ BMI, WC, BP, TG, glucose in both groups; ↓ HDL-C in intervention group; Risk of bias, High. Row 6: Author (Year), Xue et al. 2021; Country, China; Population, Adults with MetS; Study design, Open-label RCT; Sample size, 50; Duration, 12 weeks; Fibre dose, 30 g/day; Fibre source, Oat bran; Primary outcomes, BMI, BP, TG, fasting glucose; Risk of bias, Some concerns.

Navigate back to Table 4..

## Table 5. Long description

The table presents data on randomized controlled trials investigating the effects of rice bran on metabolic outcomes. It includes seven columns: Author (Year), Country, Population, Study design, Sample size, Duration, Dose, Fibre source, Primary outcomes, and Risk of bias. The table has six rows, each representing a different study. Row 1: Mahdavi-Roshan et al. 2024, Iran, Adults with MetS, Open-label RCT, 50, 8 weeks, 30 g/day, Rice bran oil, Lipids, HOMA-IR, oxidative stress, Moderate. Row 2: Lin et al. 2020, China/Taiwan, Adults (mixed metabolic status), Randomised trial, 45, 8 weeks, 40 g/day, Refined rice bran, WC, BP, glucose, HbA1c, TG, Not clearly reported. Row 3: Saphyakhajorn et al. 2022, Thailand, Overweight/obese adults, Double-blind RCT, 61, 12 weeks, 30 g/day, Defatted rice bran, HbA1c, lipids, BP, Low. Row 4: Prasertsri et al. 2022, Thailand, Older adults, RCT, 35, Several weeks, 1-2 g/day, Rice bran oil, Oxidative stress, CVD markers, Not clearly reported. Row 5: Ng et al. 2025, New Zealand, Healthy adults, Crossover RCT, 56, 4 weeks per phase (2-week washout), 3 slices/day (females) or 4 slices/day (males), Rice bran bread, Gut microbiota composition, transit time, cardiovascular risk, gut discomfort, Some concerns.

Navigate back to Table 5..

## Table 6. Long description

The table presents a summary of studies on unripe banana flour and metabolic outcomes, focusing on research published between 2014 and 2025. It includes randomized controlled trials (RCTs) and non-randomized studies. The table has 5 columns and 4 rows, with the following headers: Author (Year), Country, Population, Study design, Sample Size, Duration, Dose, Fibre Source, Primary Outcomes, and Risk of Bias. Row 1: Sardá et al. 2016, Brazil, Healthy adults, Double-blind RCT, 22, 6 weeks, ~8 g/dose (3x/week), Unripe banana flour (RS2), Satiety, insulin sensitivity, Low. Row 2: Silva et al. 2014, Brazil, Women with MetS, Intervention trial, 25, 45 days, 20 g/day, Green banana flour, BP, fasting glucose, Some concerns. Row 3: Costa et al. 2019, Brazil, Pre-diabetes/T2D, RCT, 113, 24 weeks, 40 g/day green banana biomass (approx. 4.5 g RS), Banana biomass, Glucose, weight, lipids, Some concerns. Row 4: de Oliveira Lomeu et al. 2021, Brazil, Overweight women, Double-blind RCT, 60, 8 weeks, Not reported, UBF + cocoa, Anthropometric, biochemical markers, Moderate.

Navigate back to Table 6..

## Table 7. Long description

A table comparing metabolic effects of different dietary fibers. The table has 3 rows and 8 columns. Column headers are: Fibre type, Glycaemic outcomes, Anthropometric outcomes, Lipid outcomes, Blood pressure, Oxidative stress / inflammation, Mechanistic interpretation, Strength of evidence. Row 1: Fibre type, Oat β-glucan (oat bran / enriched foods), Rice bran (defatted, refined, and oil forms), Unripe banana flour (resistant starch, RS2). Row 2: Glycaemic outcomes, Reductions in postprandial glucose responses are consistently reported, with smaller and less consistent effects on fasting glucose and HbA1c across longer interventions, Modest reductions in fasting glucose and HbA1c reported in some trials; findings are variable across populations, Improvements in fasting glucose and insulin sensitivity indices (e.g. HOMA-IR); postprandial responses show modest attenuation. Row 3: Anthropometric outcomes, Modest reductions in body weight and waist circumference, primarily in studies combined with dietary modification, Minimal or no consistent effects on body weight; small reductions in waist circumference reported in some studies, Modest reductions in appetite and, in some studies, body weight or waist circumference. Row 4: Lipid outcomes, Reductions in total cholesterol and LDL-C are consistently observed; effects on HDL-C and TGs are variable, Improvements in total cholesterol and LDL-C, with some increases in HDL-C, particularly in rice bran oil interventions, Effects on lipid profile are generally small and inconsistent. Row 5: Blood pressure, Small reductions in SBP/DBP reported in several trials, Small but significant reductions in blood pressure reported in several studies, Limited and population-specific effects on blood pressure. Row 6: Oxidative stress / inflammation, Limited and inconsistent effects on inflammatory markers; some studies report microbiota modulation, More consistent improvements in oxidative stress markers (e.g. MDA, antioxidant capacity); limited effects on CRP, Evidence suggests modulation of satiety hormones (e.g. PYY, GLP-1); limited data on inflammatory markers. Row 7: Mechanistic interpretation, Increased intestinal viscosity delays gastric emptying and glucose absorption; bile acid binding contributes to cholesterol lowering; partial fermentation may contribute to SCFA production, Combined effects of dietary fibre and bioactive compounds (e.g. γ-oryzanol, tocotrienols, phytosterols) influencing lipid metabolism, oxidative stress, and inflammation; limited contribution of fermentation relative to other fibres, Acute glycaemic effects are primarily related to reduced digestibility and delayed carbohydrate absorption, while longer-term improvements in insulin sensitivity are mediated by colonic fermentation and SCFA production. Row 8: Strength of evidence, n ≈ 6 RCTs; consistency: high for postprandial glycaemia and LDL-C; moderate for other outcomes, n ≈ 5 RCTs; consistency: moderate for lipid and BP outcomes; mixed for glycaemia, n = 3 RCTs (+1 non-randomised study); consistency: moderate for insulin sensitivity and satiety; limited for other outcomes.

Navigate back to Table 7..

## Figure 1. Long description

A flowchart illustrating the process of identifying new studies via databases and registers. The process begins with the identification of records from databases (n = 602) and registers (n = 0). Duplicate records (n = 146) are removed before screening. The remaining records (n = 456) are screened, and 412 records are excluded after title/abstract screening. Reports sought for retrieval total 44, with none not retrieved. Full text is assessed for eligibility for these 44 reports. After full-text assessment, 29 reports are excluded due to reasons such as unavailable full text, wrong setting, ineligible study design, insufficient metabolic outcomes, and ongoing trials. Finally, 15 studies are included in the review.

Navigate back to Figure 1..
